# Workflows to automate covariate-adaptive randomization in REDCap via data entry triggers

**DOI:** 10.1093/jamiaopen/ooaf110

**Published:** 2025-10-01

**Authors:** Jacob M Schauer, Marc O Broxton, Luke V Rasmussen, Gregory Swann, Michael E Newcomb, Jody D Ciolino

**Affiliations:** Division of Biostatistics and Informatics, Feinberg School of Medicine, Northwestern University, Chicago, IL 60611, United States; Information Technology, Feinberg School of Medicine, Northwestern University, Chicago, IL 60611, United States; Department of Preventive Medicine, Feinberg School of Medicine, Northwestern University, Chicago, IL 60611, United States; Department of Medical Social Sciences, Feinberg School of Medicine, Northwestern University, Chicago, IL 60611, United States; Department of Medical Social Sciences, Feinberg School of Medicine, Northwestern University, Chicago, IL 60611, United States; Division of Biostatistics and Informatics, Feinberg School of Medicine, Northwestern University, Chicago, IL 60611, United States

**Keywords:** RCT, covariate-adaptive randomization, minimal sufficient balance, REDCap

## Abstract

**Objective:**

Covariate-adaptive randomization algorithms (CARAs) can reduce covariate imbalance in randomized controlled trials (RCTs), but a lack of integration into Research Electronic Data Capture (REDCap) has limited their use. We developed a software pipeline to seamlessly integrate CARAs into REDCap as part of the all2GETHER study, a 2-armed RCT concerning HIV prevention.

**Materials and Methods:**

Leveraging REDCap’s Data Entry Trigger and a separate server, we implemented software in PHP and R to automate randomizations for all2GETHER. Randomizations were triggered by saving a specific REDCap form and were automatically communicated to unblinded study personnel.

**Results:**

Study arms were highly comparable, with differences across covariates characterized by Cohen’s *d* = 0.003 for continuous variables and risk differences <2.4% for categorical/binary variables.

**Conclusions:**

Our pipeline proved effective at reducing covariate imbalance with minimal additional effort for study personnel.

**Discussion:**

This pipeline is reproducible and could be used by other RCTs that collect data via REDCap.

## Introduction

Comparability of study arms in randomized controlled trials (RCTs) is crucial for interpreting trial results.^1–4^ However, accidental bias, which occurs when study arms markedly differ on important pre-randomization covariates (referred to as *covariate imbalance*) is a well-documented limitation of simple randomization.^1^^,^^5^ Covariate-adaptive randomization algorithms (CARAs) reduce the likelihood of accidental bias by considering current covariate imbalance (ie, on already-randomized participants) and new participants’ covariates when randomizing.^6^^,^^7^ Most modern applications involve randomizing to the study arm that would reduce covariate imbalance according to pre-specified balance criteria with a greater than 50% probability.^8–10^ Approaches to randomization, such as the minimization^6^^,^^7^ or minimal sufficient balance (MSB)^8^ algorithms, can reduce covariate imbalance and increase the power of analyses.^11^^,^^12^

Despite the advantages, the use of CARAs has lagged. Reviews of published RCTs found that 11%-12% of RCTs used such algorithms, while nearly 70% of trials report using stratified randomization, which randomizes participants within subgroups delineated by pre-selected covariates.^13^ Compared to stratified randomization, CARAs have the capacity to handle a greater number of covariates and potential to induce stronger covariate balance.^8^^,^^14^^,^^15^ Among the barriers to their broader use are their complexity and limited software options.^5^^,^^16^ Using CARAs requires careful consideration about how covariate data validation and randomization fit into study workflows. Moreover, Research Electronic Data Capture (REDCap),^17^^,^^18^ which has become the *de facto* data capture platform for many academic research centers, did not offer modules for CARAs until version 14.7. Researchers using CARAs often must write their own study-specific code files and ensure study personnel execute those scripts with every new randomization.

In this article, we describe automation of a specific CARA, the MSB algorithm, for use with REDCap as part of an RCT, the all2GETHER trial, which recently concluded recruitment.^19^ In the following sections, we outline the platform built for all2GETHER, describe interim results, and consider how other trials may leverage this framework.

## Methods

all2GETHER is a relationship education and HIV prevention program designed for gay, bisexual, queer, and transgender and non-binary people who have sex with cisgender men.^20^^,^^21^ In its current iteration, all2GETHER consists of a web app with modules containing videos and interactive activities. For partnered dyads, it also includes dyadic relationship skills coaching sessions covering topics like conflict resolution and relationship agreements.

We designed a 2-armed RCT to compare the all2GETHER intervention with a control condition in which participants are provided with HIV prevention resources from the Centers for Disease Control and Prevention (CDC).^19^ The trial aimed to recruit *N* = 5000 participants to ensure 80% power to detect a 36% risk reduction in HIV incidence (primary endpoint). Participants in the trial were 16-34 years-old and confirmed HIV-negative; they were able to engage in the trial on their own (ie, if they were single or their partner does not wish to participate) or as part of a partnered dyad. Single individuals and dyads were the unit of randomization, so both partners of a dyad were randomized to the same study arm and attended coaching sessions together. Only the prinicpal invesigator (PI) was blinded to random assignment. The protocol described applying MSB to participation status (single, partnered but participating alone, dyad), age, race/ethnicity, gender identity, and PrEP usage at baseline. Test results for rectal and urethral sexually transmitted infections at baseline were added to the algorithm in June 2023 in response to emerging imbalance.

The trial used Northwestern University’s instance of REDCap (v14.5) for data collection and storage, which did not support MSB randomization at the time of study launch (2022). To integrate MSB into study workflows at scale (*N* = 5000 target recruitment), we constructed an automated process that leverages REDCap’s Data Entry Trigger (DET) and application programming interface (API). This process runs automatically simply by saving a specific “Randomization” form in the REDCap project and returns a random assignment to unblinded study staff in seconds. All relevant code is available in a GitHub repository^22^ and additional technical details are outlined in Appendix A1.

**Figure 1.**
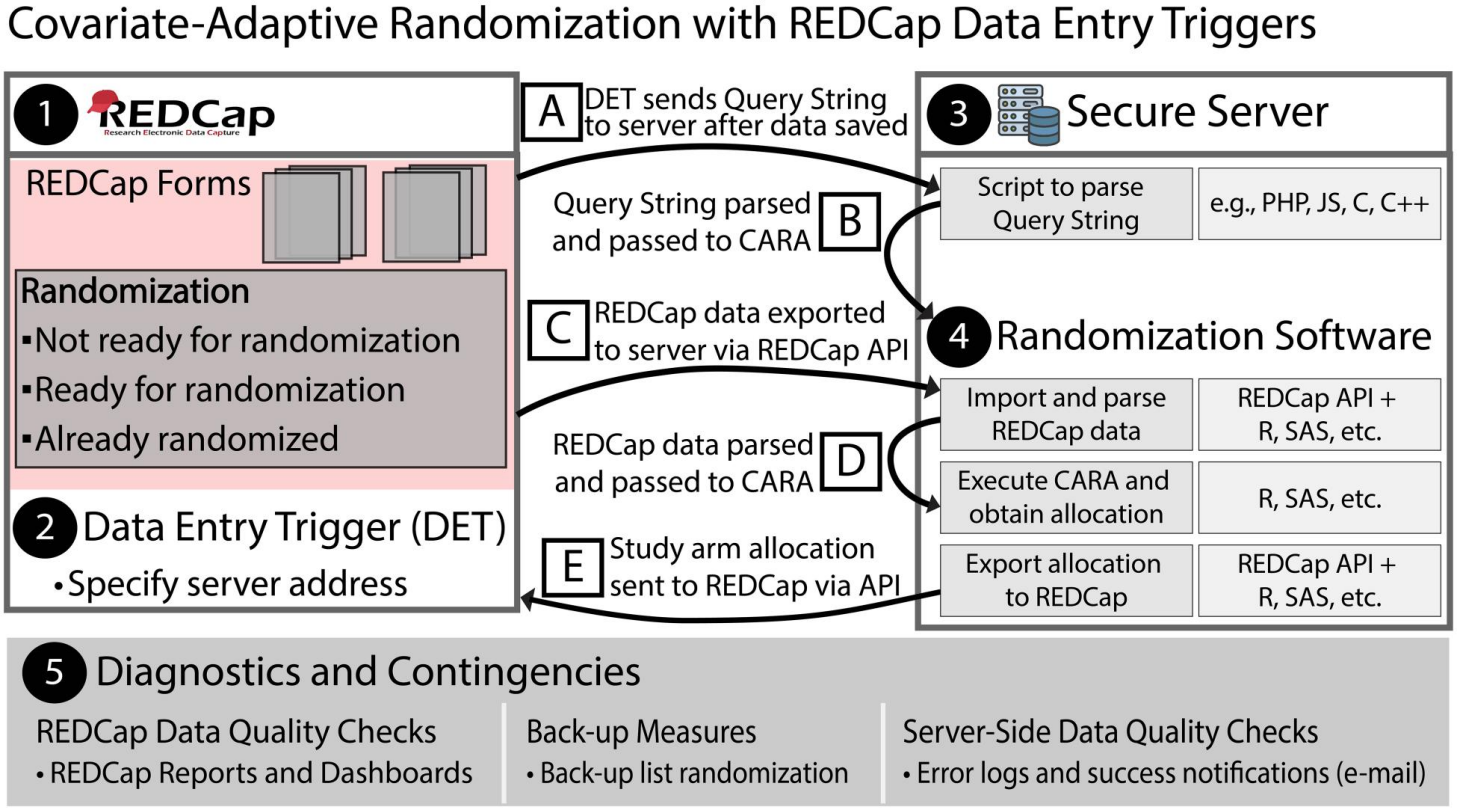
Automated Covariate-Adaptive Randomization with REDCap Data Entry Triggers.

We map the overall framework in Figure 1, which has the following core elements: (1) REDCap project containing a dedicated form indicating whether a participant should be randomized, (2) DET, (3) secure server on which to execute MSB, (4) software that implements MSB, and (5) diagnostics for quality assurance and failsafe contingencies. Working across these core elements are a series of processes (A-E) described in detail below. Critically, these processes involve verifying that randomization is required, automatically executing MSB if so, and documenting the results.

### Automated workflow components

1. *REDCap project with “Randomization” form.* Participant covariate information is collected prior to randomization and stored in the all2GETHER REDCap database. In addition, the database includes a form called “Randomization” (Figure 1) containing an item that indicates whether a participant is ready to be randomized: the *randomization indicator*. Covariate information can be collected in various REDCap forms, but we found that collating covariates in the Randomization form via calculated fields was a useful strategy. This allowed study staff to validate covariate information prior to randomization.
2. *Data-Entry Trigger.* The DET is a REDCap feature that allows users to specify a server that will be notified whenever data are entered or changed in a REDCap project. When a project record is modified, either by adding new data or modifying existing data, the DET sends an HTTP POST request with key-value pairs as part of the query string (QS) to that server containing information including the project ID, record ID, and instrument modified.
3. *Secure server.* To enable the processing of HTTP requests, we used a virtual machine running Apache web server (v2.4.37) configured for TLS1.2 and PHP-FPM (PHP FastCGI Process Manager). The server undergoes regular patching to maintain updates and security.
4. *MSB software implementation*. Our server contains software to listen for HTTP POST requests and parse the QS (Process B), format REDCap data pulled to the server via API (Process C), run the MSB algorithm (Process D), and return relevant information (including the new randomization) to REDCap (Process E). To “listen” for HTTP POST requests, our server contains a PHP script (v8.2.13) that parses the QS from the DET. The remainder of the processes are coded in the R programming language (v4.4.1). Each of these scripts automatically trigger additional steps in the randomization process. For instance, if the QS indicates a randomization is potentially required, the PHP script automatically calls R scripts to do so. Because all steps of MSB execution occur remotely and automatically on a secure server, they can be blinded to relevant study personnel.
5. *Diagnostics and contingencies*. Randomization success or failure is documented on logs kept on the server, along with reasons for failure (including software or data entry errors). Attributes of randomization, including the covariate imbalances identified by the MSB algorithm, are documented both on the server and in REDCap fields, which can be summarized via REDCap Report Dashboards. To handle server outages or other communication disruptions, a static randomization list was pre-generated as a back-up and is accessible to unblinded study personnel; in the event of an outage, a random allocation can be identified from this list and this allocation will inform future runs of the algorithm when the server is back online.

### Automated workflow processes

- *DET sends a Query String as POST request to secure server*. When data are saved or modified in REDCap, the DET sends a QS to the designated server. This QS contains information about the REDCap project ID, record ID, and instrument modified.
- *Software on server parses QS*. The PHP script identifies if the HTTP POST request comes from the correct REDCap project and if the “Randomization” form was modified, indicating randomization may be needed. In such cases, this software automatically triggers relevant actions to execute MSB (C-E below).
- *Software on server processes REDCap data*. If the DET QS indicates a participant is potentially ready for randomization (Process B), this can be verified by assessing extant REDCap data. An R script extracts data to the server (using REDCap API) and identifies if the record identified in the QS has yet to be randomized and the *randomization indicator* (Component 1) signifies they are ready to be randomized. Because MSB, like all CARAs, requires covariate data on both already-randomized and yet-to-be-randomized participants, the code identifies already-randomized participants as those with existing study arm allocations in REDCap and extracts and formats their covariate information. With these data, the R script then initiates randomization (Process D).
- *MSB software parses REDCap data and generates allocation*. The R software pipeline extracts covariate data on participants identified in Processes (B-C) who either are ready to be or have already been randomized. These data are fed into the MSB algorithm, which returns the new study allocation, randomization probability, and balance criteria that informed the randomization.
- *New random allocation passed to REDCap*. The output in Process (D), for which there exist fields within REDCap, is sent back into REDCap via the API. REDCap User Rights and Data Access Groups (DAGs) are used to ensure proper blinding. A REDCap Report dashboard is used to track allocations, probabilities, and balance criteria.

## Results

Enrollment for the trial closed in March 2025 after randomizing 2086 participants (1088 to all2GETHER, 998 to control). Automated randomization occurred with minimal server interruptions; the back-up randomization list was consulted for 18 allocations. All other 2068 randomizations were run when study staff saved the Randomization form, with each randomization taking only seconds. Statistical theory^1^^,^^2^ and research guidance^23–26^ suggest that threats to validity are minimal when covariate imbalance is no worse than a standardized difference between arms of *d* = 0.1 or a risk difference of 10%. Standardized differences^24^ for covariates in all2GETHER are reported in Table 1, with the largest being *d* = 0.052 for PREP use (absolute risk difference = 2.2%).

**Table 1.** Standardized differences for covariates used in the all2GETHER trial.

Covariate	Status	Limited participation	Race/ethnicity	GNC/trans	PrEP use	STIs	Age
**Standardized difference**	0.005	0.020	0.011	0.019	0.052	0.049	0.003

Abbreviations: GNC, gender nonconforming; PrEP, pre-exposure prophylaxis; STI, sexually transmitted infection.

## Discussion

While developing the automated pipeline for all2GETHER required collaborative frontend investment, it has proven robust and effective, eliminating delays and reducing the operational complexity associated with manual algorithm execution. Success hinged on key communication among team members, including about server patching, potential outages, and database updates. Critically, as the pipeline involves pulling and formatting covariate information from REDCap, changes relevant REDCap fields (eg, adding a new racial or gender category) must be reflected in server-side software. However, statistical guidance on CARAs underscores the purposeful selection of covariates that are highly prognostic of outcomes^5^^,^^11^^,^^16^^,^^27^ and such variables have seldom been the subject of revision in REDCap for all2GETHER.

We describe the workflow here and provide code and instructions in a related GitHub repository^22^ with the hope of reducing barriers to using CARAs in RCTs in settings using REDCap. Figure 1 can be considered a general strategy; alternate CARAs can be implemented (eg, minimization) within this framework, and the code can be modified to include participant covariates relevant for any given study and the processes and components described above can be tailored to fit research objectives and day-to-day demands. Because the entire pipeline is designed to occur automatically on a secure server, it is possible to account for various blinding criteria within this framework. Though we attempt to provide suggestions on the structure of these core elements, we note that their actual implementation will almost certainly vary across research efforts. This pipeline is currently in use for the Effectiveness of Two Aspirin Doses for Prevention of Hypertensive Disorders of Pregnancy: ASPIRIN TRIAL (clinicaltrials.gov ID: NCT06468202), while elements of it are underpinning the randomization process of the Liver Cirrhosis Network Rosuvastatin Efficacy and Safety for Cirrhosis in the United States (LCN RESCU) trial (NCT05832229).

Though not available at all institutions (including Northwestern University as of this writing), the latest instance of REDCap includes advanced randomization features that can incorporate CARAs without the need for external servers.^28^ Although specific implementations, including of MSB, are yet to be completed, we note that this increased flexibility would greatly simplify this framework, potentially eliminating the need for the DET or even a separate secure server. Our overall pipeline remains an option for researchers without access to the newest instance, while our implementation of MSB in the R programming language may help facilitate implementation within the latest randomization module.

## Supplementary Material

ooaf110_Supplementary_Data

## Data Availability

Data are available upon request. Software described in this article are available at the linked GitHub repository.

## References

[1] Altman DG. Comparability of randomised groups. J R Stat Soc Ser D Stat. 1985;34:125-136.

[2] Senn SJ. Covariate imbalance and random allocation in clinical trials. Stat Med. 1989;8:467-475. 10.1002/sim.4780080410 2727470

[3] Moerbeek M , van Schie S. How large are the consequences of covariate imbalance in cluster randomized trials: a simulation study with a continuous outcome and a binary covariate at the cluster level. BMC Med Res Methodol. 2016;16:79. 10.1186/s12874-016-0182-7 27401771 PMC4939594

[4] Gail MH , Wieand S, Piantadosi S. Biased estimates of treatment effect in randomized experiments with nonlinear regressions and omitted covariates. Biometrika. 1984;71:431-444. 10.1093/biomet/71.3.431

[5] Rosenberger WF , Lachin JM. Randomization in Clinical Trials: Theory and Practice. Wiley Series in Probability and Statistics. John Wiley & Sons, Inc.; 2016.

[6] Pocock SJ , Simon R. Sequential treatment assignment with balancing for prognostic factors in the controlled clinical trial. Biometrics. 1975;31:103-115. 1100130

[7] Taves DR. Minimization: a new method of assigning patients to treatment and control groups. Clin Pharmacol Ther. 1974;15:443-453. 10.1002/cpt1974155443 4597226

[8] Zhao W , Hill MD, Palesch Y. Minimal sufficient balance-a new strategy to balance baseline covariates and preserve randomness of treatment allocation. Stat Methods Med Res. 2015;24:989-1002. 10.1177/0962280212436447 22287602 PMC3474894

[9] Hofmeijer J , Anema PC, van der Tweel I. New algorithm for treatment allocation reduced selection bias and loss of power in small trials. J Clin Epidemiol. 2008;61:119-124. 10.1016/j.jclinepi.2007.04.002 18177784

[10] VanderWeele TJ , Vansteelandt S. Mediation analysis with multiple mediators. Epidemiol Methods. 2014;2:95-115. 10.1515/em-2012-0010 25580377 PMC4287269

[11] Lauzon SD , Zhao W, Nietert PJ, Ciolino JD, Hill MD, Ramakrishnan V. Impact of minimal sufficient balance, minimization, and stratified permuted blocks on bias and power in the estimation of treatment effect in sequential clinical trials with a binary endpoint. Stat Methods Med Res. 2022;31:184-204. 10.1177/09622802211055856 34841963 PMC9026574

[12] Lauzon SD , Ramakrishnan V, Nietert PJ, Ciolino JD, Hill MD, Zhao W. Statistical properties of minimal sufficient balance and minimization as methods for controlling baseline covariate imbalance at the design stage of sequential clinical trials. Stat Med. 2020;39:2506-2517. 10.1002/sim.8552 32363614 PMC7462097

[13] Ciolino JD , Palac HL, Yang A, Vaca M, Belli HM. Ideal vs real: a systematic review on handling covariates in randomized controlled trials. BMC Med Res Methodol. 2019;19:136-136. 10.1186/s12874-019-0787-8 31269898 PMC6610785

[14] Kang M , Ragan BG, Park JH. Issues in outcomes research: an overview of randomization techniques for clinical trials. J Athl Train. 2008;43:215-221. 10.4085/1062-6050-43.2.215 18345348 PMC2267325

[15] Scott NW , McPherson GC, Ramsay CR, Campbell MK. The method of minimization for allocation to clinical trials. a review. Control Clin Trials. 2002;23:662-674. 10.1016/s0197-2456(02)00242-8 12505244

[16] Lin Y , Zhu M, Su Z. The pursuit of balance: an overview of covariate-adaptive randomization techniques in clinical trials. Contemp Clin Trials. 2015;45:21-25. 10.1016/j.cct.2015.07.011 26244705

[17] Harris PA , Taylor R, Thielke R, Payne J, Gonzalez N, Conde JG. Research electronic data capture (REDCap)—a metadata-driven methodology and workflow process for providing translational research informatics support. J Biomed Inform. 2009;42:377-381. 10.1016/j.jbi.2008.08.010 18929686 PMC2700030

[18] Harris PA , Taylor R, Minor BL, et al; REDCap Consortium. The REDCap consortium: Building an international community of software platform partners. J Biomed Inform. 2019;95:103208. 10.1016/j.jbi.2019.103208 31078660 PMC7254481

[19] Newcomb ME. Effectiveness of Relationship Education for Reducing HIV Incidence Among SGM (all2GETHER). ClinicalTrials.gov identifier: NCT05678556. 2024. Accessed April 21, 2025. https://clinicaltrials.gov/study/NCT05678556

[20] Newcomb ME , Swann G, Macapagal K, Sarno EL, Whitton SW, Mustanski B. Biomedical and behavioral outcomes of 2GETHER: a randomized controlled trial of a telehealth HIV prevention program for young male couples. J Consult Clin Psychol. 2023;91:505-520. 10.1037/ccp0000823 37141032 PMC10729837

[21] Newcomb ME , Sarno EL, Bettin E, et al Protocol for an attention-matched randomized controlled trial of 2GETHER: a relationship education and HIV prevention program for young male couples. Trials. 2022;23:514. 10.1186/s13063-022-06457-9 35725624 PMC9207885

[22] Schauer JM. REDCap Minimal Sufficient Balance Integration. Accessed April 14, 2025. https://github.com/j3schaue/redcap_det_msb

[23] Yang S , Starks MA, Hernandez AF, et al Impact of baseline covariate imbalance on bias in treatment effect estimation in cluster randomized trials: race as an example. Contemp Clin Trials. 2020;88:105775. 10.1016/j.cct.2019.04.016 31228563 PMC8337048

[24] Yang D , Dalton JE. *A Unified Approach to Measuring the Effect Size Between Two Groups Using SAS*. Vol. 335. SAS Global Forum; 2012:1-6.

[25] Austin PC. Balance diagnostics for comparing the distribution of baseline covariates between treatment groups in propensity-score matched samples. Stat Med. 2009;28:3083-3107. 10.1002/sim.3697 19757444 PMC3472075

[26] Normand ST , Landrum MB, Guadagnoli E, et al Validating recommendations for coronary angiography following acute myocardial infarction in the elderly: a matched analysis using propensity scores. J Clin Epidemiol. 2001;54:387-398. 10.1016/s0895-4356(00)00321-8 11297888

[27] Proschan MA , Barreiro-Gomez J, Taylor F. Statistical Thinking in Clinical Trials. 1st ed. Chapman & Hall/CRC Biostatistics Series. CRC Press, Taylor & Francis Group; 2022.

[28] REDCap Con 2024: Randomization 2.0. Accessed March 18, 2025. https://mcri.figshare.com/articles/presentation/REDCap_Con_2024_Randomization_2_0/26950888, 10.25374/MCRI.26950888.v1

