# Supplementary material for: Workflows to automate covariate-adaptive randomization in REDCap via data entry triggers
Source: JAMIA Open. 2025 Oct 1;8(5):ooaf110. doi: 10.1093/jamiaopen/ooaf110 (PMC12486239; doi:10.1093/jamiaopen/ooaf110)
Supplement: ooaf110_Supplementary_Data [file ooaf110_supplementary_data.docx]

Appendix

# Software and Server Configuration

In this section, we detail the software and server setup required for all2GETHER, which used the minimal sufficient balance (MSB) algorithm for randomization, and provide some suggestions for implementation. REDCap’s Data Entry Trigger (DET) requires the URL of a server endpoint that can receive HTTP POST requests when a data entry or update event occurs. These POST requests contain URL-encoded key-value pairs (similar to form submissions) describing the REDCap event (e.g., project ID, record ID, event name, instrument, etc.); this contains components of the Query String (QS) referred to in the main manuscript. The DET target server must be accessible from the REDCap server over TCP port 443, as the communication uses HTTPS (TLS encryption). The DET server can be any system capable of accepting and processing application/x-www-form-urlencoded POST requests over HTTPS. Similarly, the REDCap API must be accessible from the DET target server over port 443 (TCP).

For all2GETHER, we used a virtual machine configured with 2 vCPUs, 4GB of RAM, and running RHEL 8.10 as operating system. To enable the processing of HTTP requests, we installed Apache web server (v2.4.37) configured for TLS1.2 and PHP-FPM (PHP FastCGI Process Manager). The PHP version currently installed is 8.2.13. To enable the execution of R scripts, we installed R version 4.4.1, along with the tidyverse and redcapAPI libraries. The server undergoes regular patching to maintain updates and security.

The installation of PHP-FPM and R are required as the software used to parse the QS and randomize participants is implemented in both PHP and R scripts. The entire software comprises the following files:

- An R script that allows users to specify REDCap fields to be used in randomization (user_specified_variables.R)
- A PHP script that listens and parses the QS (index.php)
- An R script that parses REDCap data to determine if a randomization is required (parse_payload.R)
- An R script that implements the MSB algorithm (apply_msb.R)
- An R script that randomizes new participants and sends the allocation to REDCap (randomize.R)

index.php

parse_payload.R

randomize.R

user_specified_variables.R

apply_msb.R

The general structure of the code is depicted in the figure above. The project variables, including a REDCap API token, project ID, and field names that correspond to the randomization indicator, study arm allocation, and relevant covariates are specified in the user_speficied_variables.R script. The general process is as follows:

1. When the DET fires, a QS is sent to the server and is parsed by index.php, which evaluates if the QS originated from the REDCap project ID of interest and if the Randomization instrument was modified.
2. If the Randomization form from the correct REDCap project was modified as determined by the QS, index.php automatically executes parse_payload.R, which pulls data from REDCap via the API and parses it to assess if the randomization indicator was selected for a new participant. If so, it separates the REDCap data into two groups: data for participants who have already been randomized and data for the new participant to be randomized. It then formats the relevant covariates and study arm fields according to rules set up in the user_specified_variables.R script.
3. If a new randomization is required, parse_payload.R automatically calls the randomize.R script, which in turn sources apply_msb.R–the implementation of the MSB algorithm. The randomize.R script takes the formatted data from parse_payload.R, runs the MSB algorithm, formats the updated data for the new participant (including the new study arm allocation) and sends that data back to REDCap via the API.

Across the R scripts, the only libraries called are dplyr, tidyr, and redcapAPI, which must be installed on the server (see above).

# all2GETHER

The all2GETHER implementation of MSB used the configuration originally described by Zhao et al.; the threshold was set to a statistically significant difference between arms at the α=0.3 level and randomization probability was set to π=70%. In addition, this implementation needed to account for the randomization of dyads as the covariates for members of the dyad may not be identical. Thus, the version of MSB used by all2GETHER therefore assesses if balance would be improved if *both* members of the dyad were randomized to all2GETHER or the control arm. Covariates initially included in the MSB algorithm included participant age, race/ethnicity, gender identity, and PrEP usage at baseline. This algorithm was later updated to include participation status of partners in a dyad (full or limited) and baseline STIs.

The all2GETHER REDCap project includes a form called “Randomization”. To protect against unintentional randomizations, this form includes spaces to verify some of the key covariates included in the MSB algorithm. In addition, it includes a specific item (outlined in red) to indicate that a record should be randomized, which serves as the *randomization readiness* *indicator* described above. The DET, which can be specified in the Project Setup tab in REDCap, was established prior to study launch in conjunction with Northwestern’s IT department.

The processes (B-E) outlined in the main manuscript were implemented in PHP and in the R programming language (see previous section). Successful randomizations are tracked both on REDCap and on the secure server. A dedicated REDCap report provides information on the date, covariate “votes”, and randomization probability for completed randomizations. On the server, metrics of imbalance, study arm, and de-identified participant characteristics are stored in a CSV file, which is automatically updated in our software pipeline.


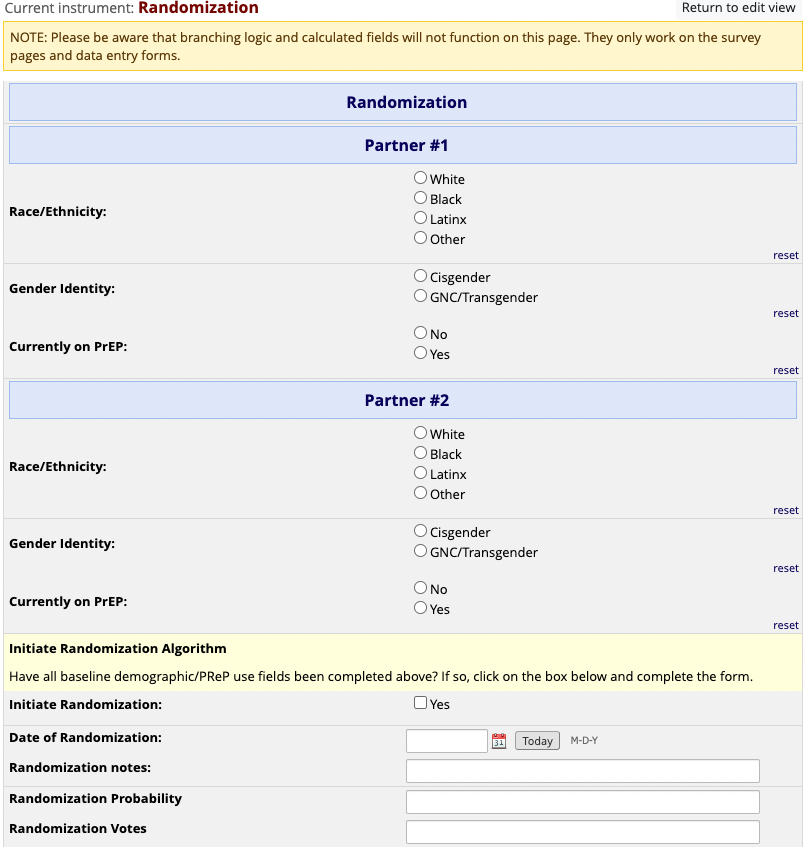


In devising processes around this software, we established a failsafe if no randomization allocation was returned to REDCap via the automated pipeline. If, after several seconds, no allocation arrived in REDCap, the Randomization form could be cleared and re-saved in attempt to re-trigger the process. If after repeated attempts, no allocation was returned, indicating a software or server error, study staff accessed a password-protected spreadsheet that contained a pre-generated randomization list. This was stored in a central location so that appropriate staff could access it in a timely and efficient manner. In the event of a backup list randomization, study staff manually indicated that a given allocation in the spreadsheet had been used and updated REDCap with that value.

At the close of enrollment, randomization occurred in the automated fashion described above and has largely preserved covariate balance, with 2,068 out of 2,086 participants being randomized via this pipeline. The bulk of the 18 randomizations that required a backup randomization list resulted from a disconnect between our initial pipeline to format data and the structure expected by REDCap when importing a new study arm via API. This led to two important improvements in the pipeline. The first was a change in the R library used to make API calls (currently redcapAPI). The second involved a broader communication across the study team regarding the stability of relevant REDCap fields used in the pipeline. To avoid scenarios where a modification to a REDCap field triggered errors when said modification was not accounted for in the software pipeline, we established a process wherein any relevant changes to REDCap involving variables used in the pipeline were updated simultaneously with the software and tested live during team meetings prior to committing changes. This has resulted in less reliance on the backup randomization list.

Overall, the algorithm has produced study arms that are sufficiently comparable as reported in the Results section of this manuscript. As an alternative approach to quantifying imbalance, we present the absolute risk difference (Table A1) between study arms on all categorical variables used in the MSB algorithm. Table A1 shows that all risk differences for each categorical variable (i.e., for each category in each of these variables) were very near zero, indicating comparability of study arms along these covariates. The largest absolute risk difference was 2.2% (PrEP use) the median absolute risk difference was 1.48% (STI prevalence), while one third of the categories in these variables exhibited an absolute risk difference of less than 1.5%.

**Table A1**. Absolute risk difference between study arms on all categorical variables used in the MSB algorithm.

| **Variable** | | **Absolute Risk Difference** |
| --- | --- | --- |
| Participant Type | Dyad | 1.05% |
|  | Partnered (Alone) | 0.90% |
|  | Single | 1.95% |
| Limited Participant | | 0.31% |
| Race | Black | 1.14% |
|  | Latinx | 2.05% |
|  | Other | 1.60% |
|  | White | 1.58% |
| GNC/Transgender | | 0.75% |
| On PrEP | | 2.24% |
| Any STI | | 1.48% |
| GNC=Gender nonconforming; PrEP=Pre-exposure prophylaxis; STI=Sexually transmitted infection | | |

# Adapting the Pipeline for Other Studies

All or part of this pipeline can be adapted to other RCTs seeking to connect REDCap to more advanced covariate-adaptive randomization processes. We have highlighted some useful processes above, including calculating or piping covariate values into the Randomization form, creation and maintenance of backup randomization lists, measures to jointly update REDCap fields and randomization software, and approaches to monitoring output. In addition to establishing such processes for an RCT, an important step in adapting the software available from the GitHub repository involves modifying the scripts described above. Initial work should focus on the following scripts:

- index.php requires an update of the project ID to ensure the ID in the script matches the REDCap project ID and the name of the Randomization form (if it is not named “Randomization”) matches the appropriate REDCap form name.
- user_specified_variables.R should be modified to specify an API token and REDCap URL, as well as to clarify the covariate fields and types, randomization allocation field, randomization indicator, and configuration for the MSB algorithm (i.e., imbalance thresholds, randomization probability, etc.).

Given the complexity of the process, it is not unrealistic and indeed has been the subject of efforts to adapt this pipeline, that the parse_payload.R script will need some form of modification.

As of this writing, we know of two RCTs that have adapted this software to conduct MSB randomization: LCN RESCU (clinicaltrials.gov identifier: NCT05832229) and ASPIRIN (NCT06468202). These studies differ in their designs, randomization requirements, as well as the elements of the pipeline they employ. ASPIRIN, which started enrolling in October 2024, has used a nearly identical setup as all2GETHER, leveraging the DET to automate randomization. This has largely involved modifying the scripts as described above and has benefitted by having seasoned statistical analysts carry out these modifications. By contrast, LCN RESCU has removed the DET automation and instead leveraged a framework that includes the parse_payload.R, randomize.R, and apply_msb.R scripts to run randomization on an as-needed basis. The reasoning for this more labor-intensive, less automated framework for LCN RESCU lies in the complexity of the trial design, which includes multiple screening visits, initial in-depth data checks for eligibility verification and accuracy, blinded drug kit numbers, drug inventory tracking, and a lead-in phase of the trial drug prior to randomization. The LCN RESCU framework, while more labor-intensive, has allowed greater control over both data quality assurance and timing of randomization to minimize randomization errors and conserve study drug inventory at the sites. LCN RESCU also illustrates that this software can function without the DET. In fact, one could plausibly adapt the software available on GitHub to run via task scheduling software.
